# Evolution of Autologous Chondrocyte Repair and Comparison to Other Cartilage Repair Techniques

**DOI:** 10.1155/2014/272481

**Published:** 2014-08-18

**Authors:** Ashvin K. Dewan, Matthew A. Gibson, Jennifer H. Elisseeff, Michael E. Trice

**Affiliations:** Department of Orthopaedic Surgery, The Johns Hopkins University, 601 N. Caroline Street, Baltimore, MD 21287, USA

## Abstract

Articular cartilage defects have been addressed using microfracture, abrasion chondroplasty, or osteochondral grafting, but these strategies do not generate tissue that adequately recapitulates native cartilage. During the past 25 years, promising new strategies using assorted scaffolds and cell sources to induce chondrocyte expansion have emerged. We reviewed the evolution of autologous chondrocyte implantation and compared it to other cartilage repair techniques. *Methods*. We searched PubMed from 1949 to 2014 for the keywords “autologous chondrocyte implantation” (ACI) and “cartilage repair” in clinical trials, meta-analyses, and review articles. We analyzed these articles, their bibliographies, our experience, and cartilage regeneration textbooks. *Results*. Microfracture, abrasion chondroplasty, osteochondral grafting, ACI, and autologous matrix-induced chondrogenesis are distinguishable by cell source (including chondrocytes and stem cells) and associated scaffolds (natural or synthetic, hydrogels or membranes). ACI seems to be as good as, if not better than, microfracture for repairing large chondral defects in a young patient's knee as evaluated by multiple clinical indices and the quality of regenerated tissue. *Conclusion*. Although there is not enough evidence to determine the best repair technique, ACI is the most established cell-based treatment for full-thickness chondral defects in young patients.

## 1. Introduction

Articular cartilage (AC) lines the surface of diarthrodial joints, provides a low-friction interface for motion, and distributes forces to underlying subchondral bone. AC lesions do not heal spontaneously and are often intractable clinical problems. Curl et al. [1] retrospectively reviewed 31516 arthroscopies, noting a 63% incidence of knee cartilage lesions and Outerbridge grade IV [2] chondral lesions in patients less than 40 years old accounting for 4% of all lesions noted at arthroscopy [1]. Advances in magnetic resonance imaging (MRI), combined with a longitudinal human trial [3], have shown that cartilage defects often progress to large, higher grade lesions over time, ultimately resulting in osteoarthritis. Arthroplasty is the definitive treatment for end-stage osteoarthritis, but its limited durability makes it better suited for older patients. Consequently, there is a need for effective methods of repairing cartilage early, which can potentially delay osteoarthritis development.

There are three predominant surgical cartilage repair paradigms. The first involves surgical access to bone marrow spaces, which promotes blood clot formation, a crude scaffold for fibrocartilaginous repair tissue produced by extravasated bone marrow stem cells (Table 1). According to Insall [4], Pridie, in the 1960s, was the first to advance this concept; subsequent iterations resulted in modern day microfracture (Table 1). The second paradigm, mosaicplasty or osteochondral autograft transfer, involves the surgical transfer of mature autologous tissue from a nonloadbearing region to a cartilage defect (Table 1) or the transfer of mature allograft tissue from a cadaveric specimen. The third and most recent paradigm, autologous chondrocyte implantation (ACI), is based on* ex vivo* expansion and subsequent chondrocyte reimplantation (Table 1). These approaches are distinguished by their cell sources and associated scaffolds. We review the evolution of autologous chondrocyte implantation, comparing it to the aforementioned cartilage repair techniques, discuss relevant comparative clinical trials, and briefly highlight some emerging new strategies for chondral repair.

## 2. Materials and Methods

An electronic literature search in PubMed (http://www.ncbi.nlm.nih.gov/pubmed) was performed to identify articles on autologous chondrocyte implantation for this review. We searched from 1949 through 2014 using the search terms “autologous chondrocyte implantation” OR “cartilage repair.” Of those 2472 articles, the 188 that were clinical trials (107 articles), meta-analyses (9 articles), or systematic review articles (72 articles) underwent abstract review. Of the 188 articles, 61 were examined in detail. For the clinical trials, the best available evidence was considered. Specifically, if Level 1 or 2 evidence was available, lower quality studies of the same cartilage repair technique were excluded from further review. All meta-analyses were reviewed. Given the large number of systematic reviews, those not published in peer-reviewed journals were excluded. Reference lists of the identified 61 articles were scrutinized to screen for other relevant articles not captured by the search. Thirteen more articles of interest were identified this way and added to our study group (*n* = 74). Subsequently, additional searches with the same entry terms were performed in the databases EMBASE and Cochrane Library databases, but no additional articles were found.

## 3. Results

### 3.1. Cell Sources for Cartilage Repair

#### 3.1.1. Chondrocytes

Chondrocytes, the predominant cell type within AC, synthesize matrix components. Because AC lacks a major vascular supply, lymphatic drainage, and nervous system innervation, chondrocytes function under avascular, anaerobic conditions, obtaining nutrients by diffusion from synovial fluid. Within AC, metabolic and morphologic profiles of deep-zone chondrocytes are distinct from those populating the superficial tangential zone. The factors responsible for this variation are unknown. Maintaining the chondrocyte phenotype with robust hyaline tissue synthesis* in vitro* during expansion for ACI is an ongoing challenge.

Given the accessibility of AC by arthroscopic surgery, native chondrocytes are a logical cell source for AC repair. The first attempts to culture chondrocytes* ex vivo* in the 1970s showed decreased production of proteoglycans and type II collagen when expanded in a monolayer [5, 6]. Although this process has been termed* dedifferentiation*, it is a misnomer and does not imply reversion to a more primitive or multipotent state.* Dedifferentiation* more accurately refers to chondrocytes with a phenotype more reminiscent of fibroblasts. Benya and Shaffer [5] seminally showed the reversibility of this process when expanded cells were cultured in a three-dimensional (3D) culture system. Many modern approaches to ACI reproduce a 3D environment by incorporating a scaffold for culturing chondrocytes.

Techniques for optimal* ex vivo* chondrocyte selection and expansion have been an area of active research. Dell'Accio et al. [7] introduced the concept of chondrocyte quality control, arguing that a more reproducible outcome of ACI can be accomplished with enriched populations of stable chondrocytes, with the greatest potential of producing cartilage* in vivo*. In the first clinical trial of ACI in 1994, Brittberg et al. [8] used anchorage-independent growth and the expression of type II collagen in agarose culture of chondrocytes to validate chondrocyte expansion. However, none of these markers predict the capacity of expanded chondrocytes to form stable cartilage tissue* in vivo*. Dell'Accio et al. [7] found that the markers COL2A1, FGFR-3, and BMP-2 were associated with a stable chondrocyte phenotype and, conversely, up-regulation of ALK-1 was negatively associated with a chondrocyte phenotype [7].

#### 3.1.2. Stem Cells

Stem cells are clonogenic and self-renewing, and they can differentiate into multiple tissue types, including cartilage. Adult stem cell and embryonic stem cells (ESCs) are active areas of cartilage repair research. The recent discovery of induced pluripotent stem (IPS) cells has also spurred new investigation.

(*1) Adult Stem Cells*. Adult stem cells contribute to the homeostatic maintenance of tissues. Two promising sources of adult stem cells are bone marrow and adipose tissue.

(*a) Bone Marrow Mesenchymal Stromal Cells (BM-MSCs)*. This common nomenclature can be confusing given the alternate names, for example, connective tissue stem cells, mesenchymal stem cells, stromal fibroblastic cells, adult multipotent mesenchymal stromal cells, and stromal stem cells. BM-MSCs are most relevant for cartilage repair in facilitating clot remodeling of microfracture, Pridie drilling, abrasion chondroplasty, and autologous matrix-induced chondrogenesis (AMIC) techniques. The notion of BM-MSCs was first suggested in 1966 by Friedenstein et al. [9], who induced osteogenesis, lipogenesis, and chondrogenesis using heterotopically transplanted bone marrow in mice; 25 years later, this trilineage potential was finally appreciated* in vitro*, and interest in mesenchymal stromal cell biology exploded. BM-MSCs comprise approximately 0.001% of mononuclear cells within human bone marrow [10]; therefore, a clot induced by penetration of the subchondral plate and extravasating bone marrow, occupying a large defect several milliliters in volume, would contain less than 100 BM-MSCs. A corresponding area of healthy AC contains approximately 10 million cells. Although microfracture and other bone-marrow-accessing techniques have produced variable clinical results [7, 11–15], the resulting tissue is fibrocartilaginous and inferior to native cartilage.

*In vitro* techniques for inducing BM-MSC chondrocyte differentiation are a popular research topic. After isolation from bone marrow aspirate, a clinically relevant amount of BM-MSCs can be expanded in culture. Minimal requisites for chondrocyte differentiation include a 3D environment, serum withdrawal, and addition of dexamethasone, vitamin C, and transforming growth factor-*β* [10]. The roles of paracrine signals from BM-MSCs in this process are incompletely understood.

(*b) Adipose-Derived MSCs*. The tissue-forming capacity of adipose-derived stem cell appears similar to that of BM-MSC, but the former is 300-fold more abundant and readily isolated. Guilak et al. [16] reviewed* in vitro* molecular and functional studies comparing chondrogenic and osteogenic potentials of adipose-derived stem cells and BM-MSCs, and although no definitive conclusions could be drawn, the potential of adipose-derived stem cells is hard to ignore.

(*2) ESCs*. ESCs are isolated from a developing embryo, and therefore ESC research is clouded in controversy. ESCs form aggregates that undergo chondrogenesis in response to multiple physical and diffusible factors [17]. Coculture with mature chondrocytes promotes ESC chondrogenesis. Hurdles to adopting ESCs include teratoma formation and host immunorejection [17, 18].

(*3) IPSs*. A recent development that might overcome ESC and adult stem cells limitations is the discovery of IPS cells, the product of somatic cell reprogramming to an embryonic-like state. Introducing a specific set of transcription factors to a terminally differentiated cell can induce reversion to a pluripotent stem cell state. IPS cells have excellent prospects for use in cartilage regeneration, but the exact techniques for directed chondrocyte differentiation have not yet been elaborated. Varghese et al. [19] have shown the feasibility of promoting mesenchymal cell differentiation and cartilage tissue production from IPS cells.

### 3.2. Scaffolds for Cartilage Repair

AC is predominantly composed of extracellular matrix (ECM), with a sparse population of chondrocytes that maintain it. Water, which comprises more than 65% of AC, is moved through the ECM by pressure gradients across the tissue. AC derives its ability to support high joint loads by the frictional resistance of the water through ECM pores. Type II collagen comprises most of AC's dry weight. The orientation of collagen bundles, along with chondrocyte organization, distinguishes AC's layers. In the last decade, basic science studies have shown the importance of paracrine signaling and cellular interaction in the development of cartilage [5, 6], and scaffolds that recapitulate native ultrastructure of ECM have emerged. Scaffolds are used as cell carriers for matrix-induced ACI (MACI; not to be confused with MACI from Genzyme Biosurgery, Cambridge, MA) and to facilitate microfracture-based repair techniques in AMIC.

Scaffold synthesis has been attempted with natural and synthetic materials. Although natural materials are attractive for their inherent complexity and biocompatibility, issues with purification, pathogen transmission, and limited mechanical properties have restricted their clinical application. Synthetic materials overcome some of these limitations but lack biologic complexity. Scaffold structures can be divided into two categories, hydrogels and membranes, based on predominant architecture; each has its own natural, synthetic, and composite materials.

#### 3.2.1. Hydrogels

Hydrogels consist of crosslinked hydrophilic polymer networks engineered to mimic cartilage's mechanical properties and can be delivered noninvasively. An attractive feature is the ability to modify the mechanical properties by crosslinking* in situ* after injection. Hydrogel crosslinking methods include light irradiation, temperature modulation, and pH change. Less crosslinked (softer) hydrogels produce dynamic loading that might favor MSC chondrogenesis [20, 21].

(*1) Natural Hydrogels*. Common, naturally derived hydrogels include alginate, agarose, chitosan, cellulose, chondroitin sulfate, and hyaluronic acid (HA). These materials are readily available, inexpensive, and easy to crosslink. Alginate and agarose were the first hydrogels used to study with chondrocytes. Hydrogels based on alginate and agarose are being piloted for clinical AMIC use (CART-PATCH, Tissue Bank of France, Mions, France). Chitosan and its chemical derivatives are obtained through the chemical modification of glycosaminoglycans found in arthropod exoskeletons. In a recent large-animal experiment, chitosan integrated well into surrounding tissue [22]. Clinically, chitosan combined with glycerol phosphate and autologous whole blood has been used in AMIC (BST-CarGel, Piramal Healthcare, Laval, Canada) [23–25]. Alginate, agarose, and chitosan are derived from nonhuman sources; immune responses have not been systemically investigated.

HA, a nonsulfated glycosaminoglycan found throughout the body, is abundant in cartilage ECM and has a 30-year track record in medical products. Uncrosslinked HA, delivered through intra-articular injection, was approved by the Food and Drug Administration in 1997 for viscosupplementation and, despite its controversial efficacy, is widely used today. HA is involved in many biologic processes, including wound healing, cell migration, and MSC differentiation. These actions are mediated, in part, through binding interactions of cell surface receptor CD44. The HA molecule length influences cellular responses. Smaller HA oligomers promote angiogenesis and subsequent bone formation; larger HA fragments are predominantly chondrogenic. To form hydrogels, HA must be chemically modified [26, 27]. Hyalograft C (Fidia Advanced Biopolymers, Abano Terme, Italy) is a form of esterified HA used clinically in MACI.

Collagen accounts for approximately 30% of all protein within the human body and has been used extensively for tissue engineering applications. Hydrogels constructed from type I and type II collagen promote cartilage formation of encapsulated cells. At the molecular level, cells interact with collagen through integrins, initiating intracellular events that promote chondrogenesis [27]. Type II collagen hydrogels enhance the* in vitro* chondrogenic differentiation of MSCs compared with type I gels; however, type II collagen degradation products can trigger cartilage breakdown* in vivo*. Two type I collagen gels are available commercially: PureCol (Glycosan Biosystems, Salt Lake City, UT) and CaReS (Arthro Kinetics, Krems, Austria).

Fibrin hydrogels have been routinely used for surgical hemostasis and tissue adhesion. They can be prepared from autologous fibrinogen and thrombin, minimizing disease transmission risk. Fibrin has inferior mechanical properties compared with other hydrogels, but it is an effective cell carrier for ACI for securing materials within cartilage defects. Fibrin glue is available commercially (Tissucol; Baxter, Vienna, Austria). Fibrin has been used to retain platelet-rich plasma in a sheep AMIC model [28]. Most recently, fibrin hydrogels have been used as a vehicle to deliver allogenic juvenile cartilage fragments; this technology (DeNovo NT; Zimmer, Inc., Warsaw, IN) is currently in clinical trials [29].

(*2) Synthetic Hydrogels*. Polyethylene glycol-diacrylate and polyvinyl alcohol are the most common synthetic hydrogels with clinical track records. Prefabricated polyvinyl alcohol hydrogels (SaluCartilage; SaluMedica, Atlanta, GA) were press-fit into debrided stage IV [2] chondral lesions; however, at 1 year, many failed to integrate with surrounding tissue [30]. Another prefabricated polyvinyl alcohol hydrogel has structural modifications to promote subchondral bone integration (Carticept Medical Inc., Alpharetta, GA). A recently developed photopolymerizable polyethylene glycol-diacrylate hydrogel, in combination with a biologic adhesive (ChonDux, Biomet, Warsaw, IN), is being investigated for AMIC in phase 2 clinical trials. Modifications to synthetic hydrogels to promote integration, integrate bioactive signals, and regulate release of soluble factors are areas under investigation.

#### 3.2.2. Membranes

(*1) Natural Membranes*. The original ACI procedure used a periosteal flap to retain transplanted chondrocytes. This procedure remains the only autologous chondrocyte technique approved by the Food and Drug Administration. Postoperative complications (e.g., pathologic flap hypertrophy), led to the development of a bilayered collagen I/III membrane substitute, a procedure known as collagen-covered ACI. This procedure has been performed extensively in Europe and has been performed “off-label” in the United States. This technology evolved into an MACI-type procedure, with culturing of expanded chondrocytes on the membrane before implantation. In its most advanced incarnation, this membrane is fabricated with a mechanically strong outer layer, an effective barrier, and an inner porous substrate for chondrocyte differentiation. Such collagen membranes are available commercially as MACI (Genzyme Biosurgery, Cambridge, MA), Maix (Matricel, Herzogenrath, Germany), or Chondro-Gide (Geistlich Biomaterials, Wolhusen, Switzerland).

(*2) Synthetic Membranes*. Synthetic aliphatic polyesters (e.g., polycaprolactone, polyglycolic acid, or polylactic acid) or their copolymers (e.g., polylactic-coglycolic) were first translated into the clinical arena as biodegradable sutures (polyglactin, vicryl). In cartilage repair, the same materials have been used in membranes. Although the degradation products (e.g., carboxylic acids and alcohols) can be toxic, degradation rates can be optimized to match their metabolic clearance to minimize toxicity.

These materials can facilitate cartilage formation and provide substantial biomechanical stability in combination with other materials. For example, the MACI graft BioSeed-C (Biotissue Technologies, Freiburg, Germany) uses a composite polylactic-coglycolic and polydioxane membrane that is infiltrated with fibrin. The Cartilage Autograft Implantation System (CAIS, DePuy Mitek, Raynham, MA) uses a copolymer membrane (35% polycaprolactone, 65% polyglycolic acid) structurally reinforced with a polydioxane mesh. Minced autologous cartilage is dispensed onto this scaffold, covered with fibrin, and held in place with degradable sutures. Nanofibrous scaffolds synthesized with these compounds using complex 3D microenvironments with maximal surface area for cell attachment that mimics ECM represent the next frontier of scaffold material science.

### 3.3. Clinical Context/Trials

Although surgical techniques penetrating the subchondral plate have existed for more than 60 years, the first clinical experience with ACI was reported in 1994 by Brittberg et al. [8]. Subsequently, interest in ACI or its subsequent iterations for cartilage defect repair has grown exponentially. In a review of 20 clinical studies of ACI, Iwasa et al. [31] found that femoral defect repairs had 60% to 90% excellent-good clinical results after 1 to 11 years.

The literature on ACI is dominated by numerous case series. Reports of patients with chondral defects treated with ACI, now with 2 to 20 years of follow-up, have reported good-excellent results [32–41]. Only a limited number of prospective, comparative trials of ACI exist. Three meta-analyses of randomized trials have shown insufficient evidence to establish the effectiveness of ACI relative to other cartilage repair methods [42–44].

#### 3.3.1. First-Generation Autologous Chondrocyte Implantation

In its first iteration, ACI involved inoculation of expanded chondrocytes under a membrane flap. Results have indicated that ACI is comparable to microfracture and mosaicplasty. Knutsen et al. [13] compared ACI with microfracture in a randomized trial of 80 patients with femoral chondral defects, noting at 2 years' follow-up no histologic differences by biopsy and equivalent clinical measures between the two groups, except for a higher Short Form-36 physical component score in the microfracture group [13]. 5 years' follow-up [12], no difference was noted between the two groups; a third of both groups showed early radiographic osteoarthritis progression.

In a randomized trial of 47 patients with chondral defects, Dozin et al. [45] compared ACI with mosaicplasty after an initial debridement 6 months previously. Interestingly, 14 patients recovered without further intervention. For the 23 remaining patients who underwent treatment, there was no difference between the two groups at 12 months' follow-up. Despite the low power of the study and large number of patients lost to follow-up, the spontaneous improvement after debridement for some was intriguing. Bentley et al. [46] compared ACI with mosaicplasty in a randomized trial of 100 patients with chondral defects. At 1-year follow-up, ACI was superior according to the Cincinnati and Stanmore scores and second-look arthroscopy. (Note: the reported clinical indices are not validated for cartilage repair.) At 10 year's follow-up in the same group of patients, Bentley et al. [47] found failure rates with ACI and mosaicplasty of 17% and 55%, respectively, and significantly better functional outcome with ACI using the same indices. In contrast, Horas et al. [48] compared 40 patients with femoral defects randomized to ACI or autologous osteochondral transplant treatment and found that no clinical difference between the two techniques was found at 2 years' follow-up. By biopsy, however, osteochondral plugs showed preserved hyaline tissue compared with a predominance of fibrocartilage in ACI.

As first-generation ACI evolved, surgeons substitute the periosteal patch with collagen membranes to avoid the morbidity of periosteum harvest and* in situ* periosteal patch hypertrophy. Gooding et al. [49] compared ACI using a periosteal path with ACI using a type I/III collagen membrane cover in a randomized trial of 68 patients with chondral defects. At 2 years' follow-up, functional outcomes were equivalent, but ACI periosteal patched grafts were often complicated by symptomatic hypertrophy.

The application of characterized ACI [7] (see Section 3.1) has been explored by multiple investigators. Van Assche et al. [15] compared it with microfracture in a trial of 67 patients and found at 2 years' follow-up no differences in patient activity levels [15] and similar functional outcome, but slower recovery initially with characterized ACI [14]. In a large multicenter randomized trial, Saris et al. [50] compared characterized ACI with microfracture in 118 patients. At 1-year follow-up, they found superior histologic evidence of characterized ACI repair but equivalent clinical outcomes. By 3 years' follow-up, the characterized ACI group had significantly better clinical outcomes; time to treatment and chondrocyte quality were associated with better outcomes [51].

The clinical outcomes of ACI in specific patient populations have been investigated. In a case series of 20 adolescent athletes with chondral defects, at a mean follow-up of 47 months, Mithöfer et al. [52] noted good-excellent clinical results, with 60% return to sport after ACI. In another case series of 45 soccer players with chondral defects, at a mean follow-up of 41 months, Mithöfer et al. [53] noted that 72% had good-excellent results, but only 33% returned to soccer after ACI. The authors concluded that younger patients who presented sooner for surgery fared better. Peterson et al. [54] evaluated ACI for osteochondritis dissecans in a cohort of 58 patients. At a mean follow-up of 5.6 years, they noted that 91% had good-excellent clinical outcomes. Niemeyer et al. [55] conducted a prospective age-matched pair analysis of 37 ACI patients 40 years old or older. At 2 years' follow-up, there was no difference in multiple clinical indices between age groups.

#### 3.3.2. Second-Generation Autologous Chondrocyte Implantation

The incorporation of a scaffold or substrate to promote chondrocyte expansion represented the next step in ACI evolution, also known as MACI. Compared with abrasive techniques, the results have been promising. In a trial of 50 patients, Višňa et al. [56] compared MACI using autologous chondrocytes cultivated in a fibrin carrier with abrasive techniques. At the 1-year follow-up, the MACI group had significantly better clinical outcomes. Basad et al. [11] compared a type I/III collagen scaffold for ACI (MACI; Genzyme Biosurgery) with microfracture in a randomized trial of 60 patients. At 2 years' follow-up, the MACI group had significantly improved cartilage repair clinical indices.

Some investigators have compared MACI to older ACI techniques. Zeifang et al. [57] compared MACI with periosteal flap technique ACI in a randomized trial of 21 patients. At 2 years' follow-up, the results were equivocal. Bartlett et al. [58] compared MACI with collagen patch technique ACI in a randomized trial of 91 patients and, at 1-year follow-up, arrived at a similar conclusion, that is, the two groups were clinically equivalent, with similar histologic grades by biopsy and hypertrophy rates.

Multiple case series for proprietary technologies have been published. Three case series using Hyalograft C with autologous chondrocytes in chondral defects have shown promising clinical results at 2 years' follow-up with low complication rates [59–61]. In a prospective nonrandomized trial of 80 patients comparing microfracture with Hyalograft C MACI, Kon et al. [62] showed that Hyalograft C had better clinical outcomes and faster return to sport at 5 years' follow-up.

Another group examined CaReS, a 3D collagen-gel-based MACI technique, for patients with femoral chondral defects [63, 64]. A recent study using MRI and the Brittberg score to compare cartilage repair by Hyalograft C and CaReS MACI techniques in 20 patients 2 years postoperatively showed comparable clinical outcome, but different repair tissue composition [65]. The preliminary experience with another MACI alternative, BioSeed-C, a bioresorbable 2-component gel-polymer scaffold embedded with autologous chondrocytes, has also been promising [66, 67].

The Histogenics NeoCart technique represents an extension of MACI-based therapies. Using a tissue bioreactor to introduce mechanical loading, a proprietary matrix is seeded with autologous chondrocytes to prepare cartilaginous tissue [68]. Preliminary results at 2 years' follow-up showed decreased pain and hyaline-like fill in chondral defects by MRI [69]. This result was recently validated in a randomized trial (30 patients) comparing NeoCart and microfracture [70].

Minced AC (Cartilage Autograft Implantation System) is another novel second-generation, unique single-stage cartilage repair technique that mixes minced autograft cartilage in a carrier gel/substrate. Clinical trials with this technology are underway [29, 71].

#### 3.3.3. Third-Generation Autologous Chondrocyte Implantation

Third-generation ACI uses allogenic tissue or autologous stem cells for cartilage regeneration, avoiding the morbidity of autogenic cartilage harvest and two surgical procedures. DeNovo ET, an off-the-shelf chondroconductive/inductive matrix with allogeneic fetal chondrocytes for implantation in chondral defects [68], uses minced juvenile allograft donor cartilage to fill chondral defects. Clinical trials are underway [29, 71]. Bekkers et al. [72, 73] are investigating a one-stage approach to ACI combining primary chondrocytes with mesenchymal stromal cells without* ex vivo* expansion before implantation. They hypothesize that mesenchymal stromal cells will help prevent existing chondrocytes from dedifferentiating and promote cartilage repair. Cole and Gomoll are currently conducting a clinical trial for another proprietary product, CARTISTEM, which uses mesenchymal stem cells from umbilical cord blood to culture a hyaluronate-based gel for one-stage implantation [71]. The clinical trials for these promising new techniques are underway.

### 3.4. Other Techniques

Benthien and Behrens [74] have advocated the use of AMIC (Table 1), an enhanced microfracture method for cartilage repair (see the AMIC discussion above). They applied autologous collagen and fibrin glue matrix-induced chondrogenesis to the treatment of focal cartilage defects of the knee. In a retrospective cohort of 27 patients (minimum 2 years' follow-up), they observed that 87% were highly satisfied, with significant improvement in multiple clinical scores and MRI evidence of defect filling [75].

Stanish et al. [25] recently published a randomized control trial comparing the repair of femoral osteochondral defects in 80 patients with microfracture or a chitosan hydrogel-based proprietary AMIC technology called BST-CarGel. At 1 year of follow-up, they noted superior lesion fill and repair tissue quality on MRI with BST-CarGel. The clinical outcome scores as measured by the Western Ontario McMaster Universities Osteoarthritis Index and Short Form-36 were equivalent.

In a prospective case series of five patients, Dhollander et al. [76] reported on the outcomes of autologous platelet-rich plasma gel-induced chondrogenesis for patellar defects. At 2 years' follow-up, all experienced clinical improvement, but MRI findings were not as favorable, showing intralesional osteophytes and subchondral bone changes [76].

Multiple other commercial products using AMIC, such as ChonDux and CART-PATCH, are in clinical trials.

## 4. Conclusion

Basic science advances have fueled the development of ACI and are promoting the development of new cartilage repair techniques. The benefit of these new strategies compared with established cartilage repair techniques is not yet established, and the promise of one-stage techniques that harness the potential of stem cells to create organized hyaline-like repair tissue* in situ* remains the elusive goal. As cartilage regeneration research matures, long-term follow-up and larger comparative trials will ultimately establish the optimal method for cartilage repair.

## Figures and Tables

**Table 1:** Summary of techniques.

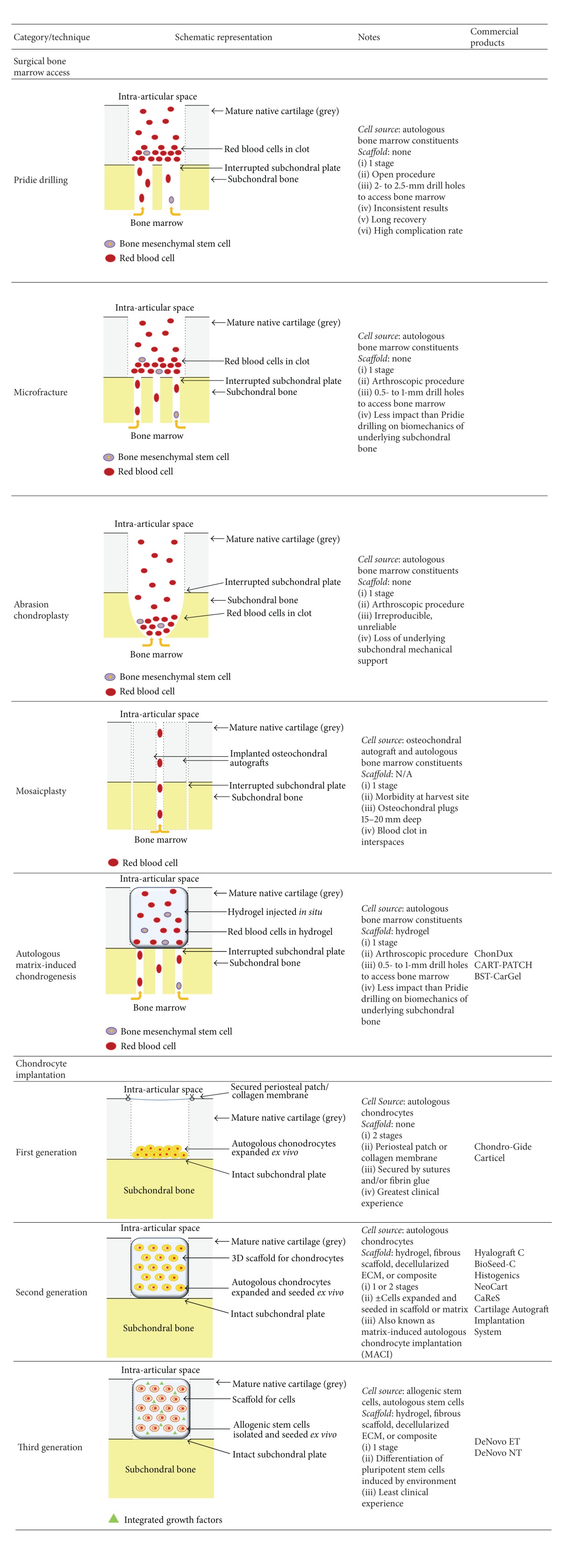

## Abbreviations

3D: Three-dimensional
ACI: Autologous chondrocyte implantation
AC: Articular cartilage
AMIC: Autologous matrix-induced chondrogenesis
BM-MSCs: Bone marrow mesenchymal stromal cells
ECM: Extracellular matrix
ESCs: Embryonic stem cells
HA: Hyaluronic acid
IPS: Induced pluripotent stem
MRI: Magnetic resonance imaging.
